# Personal distress as a mediator between self-esteem, self-efficacy, loneliness and problematic video gaming in female and male emerging adult gamers

**DOI:** 10.1371/journal.pone.0226213

**Published:** 2019-12-10

**Authors:** Andrzej Cudo, Natalia Kopiś, Emilia Zabielska-Mendyk

**Affiliations:** Department of Experimental Psychology, The John Paul II Catholic University of Lublin, Lublin, Poland; International Telematic University Uninettuno, ITALY

## Abstract

The aim of our research was to investigate the predictors of Problematic Video Gaming (PVG) in emerging adulthood. From among the factors which were considered significant in previous studies, we decided to include the following in our research: empathy, self-esteem, self-efficacy and loneliness. Additionally, we wanted to examine which predictors have a direct or indirect effect on PVG in female and male emerging adult gamers group. Including a sample of 370 video game players (201 female gamers) aged 18–30 years (M = 21.66 years, SD = 2.83) participated in this study and were asked to complete self-report measures. The questionnaires included: Problem Videogame Playing Questionnaire, The Interpersonal Reactivity Index, Rosenberg Self-Esteem Scale, De Jong Gierveld Loneliness Scale, General Self-Efficacy Scale. Our results indicate that empathy dimension associated with personal distress, the time spent playing computer games per week is directly associated with PVG. Also, there were found full mediation between self-esteem, loneliness, self-efficacy and PVG via personal distress in emerging adult male gamers group. In emerging adult female gamers group personal distress fully mediated relation between self-esteem, self-efficacy and PVG. Our findings indicate that the time spent playing video games, as well as personal distress as a function of self-esteem, loneliness and self-efficacy, are predictors of problematic video gaming. Additionally, our results may lead to a better understanding of PVG among emerging adults. In particular, they may point to the importance of personal distress in relation to PVG during emerging adulthood, which is a developmental stage of many changes in social and professional life.

## Introduction

The last decades have abounded in the development of new technologies like computers, the Internet or smartphones, which have contributed to improvements in the life of an average person. Over time, computers have become an almost indispensable part of human life. Currently, they are not only a tool of work but also of entertainment. Therefore, more and more people use them to play video games, both offline and online [1,2]. Gaming allows for virtual communication with others and achieving success in undertaking complex missions together. Players have the power to influence their gaming environment and carry out transactions with virtual or real assets [3]. A common motivation for playing video games is to relax from work or to escape boredom [4,5]. Despite the fact that computer games promote pleasant spending of time and may development of cognitive functions [6] some studies showed that long-term computer game playing could result in problematic video gaming (PVG) [7]. PVG has become a major social problem and it is described in DSM-5, Section III as the “Internet Gaming Disorder” [8]. Gaming disorder has also been included in the latest edition of the International Classification of Diseases (ICD-11) defines the behavior as follows: “Gaming disorder is characterized by a pattern of persistent or recurrent gaming behavior (…) manifested by: 1) impaired control over gaming (…); 2) increasing priority given to gaming to the extent that gaming takes precedence over other life interests and daily activities; and 3) continuation or escalation of gaming despite the occurrence of negative consequences”. It should be noted that PVG and problematic Internet use are not the same [9]. PVG may be treated as a subtype of specific Internet-use disorder [10]. In this type of Internet addiction, in contrast to general Internet-use disorder, the users search for specific material on the Internet, e.g. pornography, games, gambling, social networking sites etc. However, this specific material (games for PVG) can also be searched for off-line. Brand et al. [10] also pointed out that predispositions of users may shape the specific pattern of their Internet use, for example, those with higher sexual expectations may be more likely to search for pornographic material.

Most of the research [11–15] reported that adolescents and emerging adults are the most vulnerable group to PVG. Ream, Elliott and Dunlap [16] showed that number of days of play and PVG had curvilinear relationships with age, more specifically they were rising through childhood and adolescence to a peak and then they were levelling off or decreasing in emerging adulthood. Consequently, in this paper, consideration is given to emerging adulthood defined as developmental stage between 18 and 29 years with evolutionary tasks and phase-specific characteristics different from both adolescence and adulthood [17–19]. There are five features distinctive for emerging adulthood: identity explorations, instability, self-focus, feeling in-between, and possibilities or optimism [18]. At this developmental stage individuals try out different possibilities in various areas of life, specifically in love, relationships, work, and ideology. Also, it is a phase when people have the fewest daily social roles and obligations to others, which in consequence may result in more self-focused way of life in this period. At this developmental stage person may have a tendency to feel being between puberty and adulthood, as well as not being teenagers or adults, but somewhere in between. Additionally, emerging adults point to a large number of opportunities in their lives and they are optimistic about their actions and decisions [17,18]. Emerging adults, as opposed to adolescents, have reached physical and sexual maturity. Also, at this developmental stage individuals are more diverse in terms of education and work, and they are increasingly independent of their parents. Compared to adults in their thirties, emerging adults have often not yet integrated the stable structure into their life, with long-term commitments to love relationships and work. They often change jobs and experiment in love life before making any long lasting decisions [17,18].

Taking into account that emerging adulthood is a developmental stage of many changes in social and professional life, individuals may experience various emotional-behavioral difficulties in relation to these changes [20]. In this context, for emerging adults as well as adolescents, problematic behaviours such as PVG or problematic Internet use may be considered as a maladaptive strategy used to cope with negative emotions and psychological/mental suffering [21–23]. For instance, Li et al. [24] showed that the relationship between stressful life events and PVG was fully mediated by avoidant coping styles in emerging adult group. However, it should be noted that the source of these negative emotions and psychological suffering may be different in both groups. In the case of adolescents, physical maturation associated with hormonal, neural, physical changes, and family functioning may be more related to negative emotions and distress than in emerging adulthood. On the other hand, emerging adults may experience negative psychological states associated with failure to find a stable job, satisfactory love relationships and the life stability which may be less felt by adolescents [17,18]. In this context, Przepiorka, Blachnio and Cudo [25] showed difference between emerging adults and adolescents in relationship between long future time perspective and problematic Internet use. More precisely, this negative relationship was significant for emerging adults but not significant for adolescents. These results may be an argument in the discussion about differences between the emerging adults and another development group such as adolescents in the context of problematic behaviors.

As regards the prevalence of PVG in emerging adulthood, Khazaal et al. [26] presented that 2.3 percent young Swiss men in their twenties revealed PVG symptoms. Additionally, based on the review by Long et al. [13] the percentage of emerging adult gamers manifesting PVG ranged from 1.5 to 4 percent in China. Cudo et al. [7] also showed that 3.6 percent of the emerging adult gamers (age from18 to 30 years) presented PVG symptoms in Poland. Mentzoni et al. [27] demonstrated that around five percent young Norwegians in their twenties may manifest PVG. Lemmens et al [12] showed that 8.5 percent of the emerging adult gamers (age from 21 to 30 years) presented PVG symptoms in Netherlands.

Additionally, the results of some studies [12,13,24,27] showed that emerging adults male gamers have more symptoms of PVG than emerging adults female gamers. For example, about five percent of Norwegian emerging adults reveal symptoms of PVG, including 9.7 percent males and 1.1 percent females [27]. Numerous studies review the differences between male and female gamers revealed that in terms of accuracy, persistence, consequences, and future prospects, many female gamers play in the same way as male gamers, without giving way to them in terms of both playing time and skill [28]. Also, the results of the other research indicate that female and male players no difference in term of PVG symptoms [7]. However, there are differences in the motivation or expectations of male and female players still noted in psychological studies [29–31]. For instance, Von Der Heiden et al. [22] showed that male gamers preferred action and strategy game genre as compared to female gamers. Other studies also confirm these differences [31,32] and research shows that action game genre is positively related to PVG [7]. Among the motives for playing, for male players, coping was a predictive of PVG, while for female players, competition was a predictor of PVG. For both gender groups, escapism was a predictor of PVG [33]. Additionally, Maroney et al. [34] found that relationship between psychosocial distress associated with depression, loneliness, social anxiety and PVG was mediated by motivations regarding gaming, such as playing video games to escape negative states and seeking to satisfy the needs of social interaction. In this context longitudinal study by Jenzer et al. [35] showed that females used social support-seeking coping strategy more often than males at baseline. Additionally, males reported a small decline in the frequency of using this strategy over time. Jenzer et al. [35] men and women are becoming increasingly diverse in terms of how they use other people to cope with stress throughout emerging adulthood. It can therefore be assumed that playing video games may be a way of dealing with stressful situations, including the search for social support-seeking. However, it may be in different form between female and male gamers.

Taking together, emerging adulthood is a developmental stage in which individuals are vulnerable to PVG. Emerging adults can use the games as a way of dealing with negative emotions and psychological sufferance. Also, search social support-seeking can be an important factor in reaching for video games. However, female and male can use games in different ways and they may show different PVG predictors.

### Self-esteem, self-efficacy, loneliness, empathy and PVG

Studies to date concerning gamers have focused to a large extent on such personality variables as self-esteem [11,22,36,37], self-efficacy [22,38] and loneliness [22,39,40] as the predictors of PVG. The findings of these studies mostly show the negative impact of PVG on gamers in social and psychological terms. Additionally, self-esteem, self-efficacy and social relationship are essential in emerging adulthood [17,18,41–44]. Given that people at this stage of development undergo many changes in their social and professional lives, high self-esteem, high self-efficacy and social support may contribute to better coping with these changes [17,18,41,43,44]. It also should be noted that deficits in emotional functionality were predictors of PVG [45]. In this context, empathy is one of the most important components of everyday emotional functioning [46]. Additionally, self-esteem, loneliness and self-efficacy were associated with empathy [47–50]. It is therefore important to understand the relationship between self-esteem, loneliness self-efficacy, empathy and PVG. Moreover, emerging adults engage in a variety of social interactions related to work and love life [17,18,41]. Therefore romantic relationships can be an important source of well-being for emerging adults [51]. However, the satisfaction of an intimate relationship is associated with empathy [52–54]. Consequently, deficits in empathy may lead to unsuccessful love relationships which in turn may eventually lead to experiencing various emotional-behavioral difficulties by individuals. In this context, PVG may be considered as a maladaptive strategy used to cope with negative emotions and psychological suffering. Taking together, it is crucial to understand the relation between self-esteem, loneliness self-efficacy, empathy and PVG in emerging adulthood.

First, Brown [55] defines self-esteem as positive and negative feelings about oneself, fundamentally based on an affective process. It has been demonstrated in research that male high school students who spend more time playing video games are also characterized by lower self-esteem in comparison with boys who spend less time playing video games [55]. However, research by Behm-Morawitz and Mastro [38] do not confirm these results in the group of female players. Additionally, Koronczai et al. [56] showed that in case of problematic Internet use, self-esteem was full mediated by depression and anxiety in female and male group. Consequently, it should be pointed out that the link between self-esteem and PVG may be, on the one hand, gender-specific and on the other hand mediated by other variables.

Secondly, according to Perlman and Peplau [57] loneliness is “the unpleasant experience that occurs when a person’s network of social relations is deficient in some important way, either quantitatively or qualitatively”. Von Der Heiden et al. [22] showed that a higher level of loneliness is associated with more symptoms of PVG. Chou and Tsai [58] presented that both female and male gamers marked escape from loneliness as one of the motivating factors for playing computer games. Additionally, the female gamers reported experiencing anxiety and loneliness due to lack of social support [59].

And thirdly, self-efficacy is defined as “the belief in one’s efficacy to exercise control over one’s functioning and events that affect one’s life” [60]. Research results show that the interactive nature of games, or the speed with which they react to every single movement of the player, motivates the gamer to continue playing. As a result, the gamer experiences himself or herself as a causative factor within the game environment [61]. Behm-Morawitz and Mastro [38] and Terlecki et al. [62] point out that female gamers felt a little less self-confident about their ability to perform the tasks of the game. Male players, on the other hand, feel more qualified and self-confident. Also, Lopez-Fernandez et al. [31] indicated that female gamers look for different things in video games than male gamers, which are not often included in game designs thereby limiting their abilities.

Apart from the above-mentioned variables, however, it is indicated that what is significant in the case of PVG is emotional functioning, whose important component is empathy [46]. In Davis’s concept, empathy is defined as a reaction of one individual to the observed experience of another [47]. This concept assumes that empathy comprises emotional, cognitive, as well as behavioral aspects, considered in the intra- and interpersonal dimension [47]. It includes the conditions preceding the occurrence of empathy, empathic reactions triggered by the observer and the effects of these processes. The model is also situationally conditioned (the intensity of the situation, the degree of similarity between the observer and the observed). Empathic behaviour and thinking ensues from the personality properties of an individual. These properties include: (1) “a tendency to spontaneously adopt the psychological point of view of others" (the Perspective Taking); (2) the ability "to assess 'other-oriented' feelings of sympathy and concern for unfortunate others" (the Empathic Concern); (3) "the self-oriented feelings of personal anxiety and unease in intense interpersonal settings" (the Personal Distress; PD) [47]. It should be noted that men have lower level on the all three dimensions of empathy than women [63,64].

The multi-dimensional scale of empathic reactions developed by Davis (the Interpersonal Reactivity Index (IRI) allows for measuring the forms of empathic reactions indicated above [47]. The results of studies to date indicate a relationship between the emotional aspect of empathy, that is PD, and addictions such as gambling addiction [65], problematic Internet use [66] and problematic smartphone use [67], in which the level of PD turned out to be higher in addicts compared to healthy controls. Individuals with a higher level of social dysfunction and lower levels of social competence achieve high levels on the scale of PD [47]. Individuals characterized by a high level of PD tend to be more socially anxious and shy, but also report low self-esteem and a strong tendency toward chronic fearfulness.

### Current study

On the basis of the results of recent research on video game playing, it should be noted that it is important to search not only for single predictors of PVG, but also for the relations between a greater number of predictors and this type of problematic behaviour [68,69]. The aim of our research was to look for predictors of PVG among the factors which were considered significant in previous studies: self-esteem, self-efficacy, loneliness and empathy, in a sample of young male and female emerging adult gamers. In addition, we wanted to see which predictors may have a direct or indirect effect on PVG. Taking into consideration the fact that PD is strongly connected with low self-esteem, and, moreover that high results on this scale correlate positively with poor social functioning (shyness and social anxiety) and that it is also strongly associated with vulnerability, uncertainty and fearfulness [47–50], it can be assumed that PD would be connected with self-efficacy and high loneliness as well, and that it will also be the dominant predictor for PVG. Consequently, we assumed that relation between self-esteem, self-efficacy and loneliness and PVG [38,58,62] would be mediated by PD (H1). Furthermore, in the light of the results of previous research about empathy and problematic behaviour [65–67], we hypothesize that there would be positive relation between PD and PVG (H2). What is more in the lights of differences between female and male emerging adult gamers [15,36], we postulate that the relation between PD and PVG would be stronger in female emerging adult gamers group than in male emerging adult gamers group (H3). And finally, we take into account the fact that there is a relationship between PVG and the time spent playing [12], as problematic behaviour is intensified with an increase in gaming time [70]. Consequently, we hypothesized that the time spent playing would be positively related of PVG (H4).

## Materials and methods

### Participants

The study was carried out on a group of 370 video game players (201 female gamers) aged 18–30 years (M = 21.66 years, SD = 2.83) selected from 789 emerging adults which completed a paper questionnaire. The criteria for inclusion of selected participants were based on whether they are currently actively playing, and more specifically, those who had declared themselves playing video games in the last year were selected for the survey. All participants were volunteers and most of the participants were university students from Lublin. The mean time spent on playing video games was 11.20 hours per week (SD = 13.49). The study was conducted in accordance with the Declaration of Helsinki. Participants were informed that their responses would be anonymous and the oral participants’ informed consent was obtained. The study protocol was approved by the Ethical Committee of the Institute of Psychology of The John Paul II Catholic University of Lublin.

### Measures

1) Problem Videogame Playing Questionnaire (PVP) [71]. It comprises 9 statements which are rated by subjects using a dichotomous scale. A greater number of positive responses provided by a subject corresponds with a stronger compulsion to play video games. However, PVG is reflected by no less than six positive answers identified in the Problem Video Game Playing Questionnaire [72]. PVP questionnaire has good clinical accuracy and is commonly applied in research on PVG [73]. The original version of the questionnaire has good psychometric properties, with Cronbach’s alpha equal 0.66. Also the present version of the questionnaire has good psychometric properties, with Cronbach’s alpha equaling 0.62. Additionally, the Internet Gaming Disorder Scale–Short-Form (IGDS9-SF) [74,75] was positively correlated with Polish version of the Problem Video Game Playing Questionnaire (r = 0.78). The original version of Problem Videogame Playing Questionnaire correlates with the frequency of gaming (r = 0.48), with mean time per session (r = 0.44) and with the longest gaming sessions (r = 0.47) [71].

2) The Interpersonal Reactivity Index (IRI) [47]. This method is a Polish adaptation and validation of Davis’s questionnaire [76]. The scale consists of 28 items which form three subscales: Empathic Concern scale contains 11 items; Personal Distress scale contains 9 items and Perspective Taking scale contains 8 items. Empathic Concern scale and Personal Distress scale are related to emotional empathy. Responses were given using 5-point Likert-type scales, ranging from 1 (does not describe me well) to 5 (describes me very well). Davis [47] reported Cronbach’s alpha levels for the subscales ranging from 0.71 to 0.77. In the Polish version of the Interpersonal Reactivity Index (IRI) Cronbach’s alpha equals 0.78 for Empathic Concern scale, 0.78 for Personal Distress scale and 0.74 for Perspective Taking [76].

3) Rosenberg Self-Esteem Scale (SES) [77] in a Polish adaptation and validation by Łaguna, Lachowicz-Tabaczek and Dzwonkowska [78]. The scale includes 10 items to which answers are given on a 4-point Likert scale, from 1—I strongly agree to 4—I strongly disagree. A higher score indicates a higher level of individual self-esteem. The Polish version of the Rosenberg Self-Esteem Scale has good psychometric properties, with Cronbach’s alpha equal 0.82.

4) De Jong Gierveld Loneliness Scale [79] in a Polish adaptation by Grygiel and colleagues [80]. The tool is composed of 11 statements to which respondents react using a five-degree Likert scale, from 1 –definitely yes, to 5 –definitely no. A higher score indicates greater feeling of loneliness. The Polish version of the questionnaire has good psychometric properties, with Cronbach’s alpha equaling 0.89. Additionally, Polish version of the questionnaire positively correlated with UCLA Loneliness Scale (r = 0.82) [80].

5) General Self-Efficacy Scale (GSES) [81] in a Polish adaptation by Schwarzer, Jerusalem and Juczyński [82]. The scale is composed of 10 statements to which respondents response using a four-degree scale, from 1 (no) to 4 (yes). Higher scores indicate higher perceived general self-efficacy. The Polish version of the questionnaire has good psychometric properties, with Cronbach’s alpha equaling 0.85.

6) A short questionnaire consisting of questions about socio-demographic status (age, gender, etc.) and the number of hours spent playing video games per week.

### Statistical analysis

Descriptive statistics are presented in the form of arithmetic means and standard deviations. Spearman’s rank correlation coefficient was applied to determine relationships between the variables. In order to examine the relationship between self-esteem, self-efficacy, loneliness, gaming time, empathy and PVG in the sample of female and male gamers, an analysis of structural equations was conducted. It was based on the maximum likelihood method, applied to estimate a structural model for PVG. Taking into account previous research [47–50], the model was developed indicating self-esteem, self-efficacy, loneliness as a predictors of empathy. Also, the relation of these variables and empathy to the PVG was considered. The following statistics were applied as measures of model fit: χ^2^, χ^2^/df, RMSEA, SRMR, GFI, CFI, NFI, TLI [83,84]. Statistically insignificant (p > 0.05) chi-square values may suggest that the proposed model fits the dataset well. The value of chi-square/df ratio is lower than 2 and suggests a good fit to the dataset. Likewise, values of RMSEA and SRMR lower than 0.05 show a good fit of the model. Values of GFI, CFI, NFI, TLI higher than 0.95 allow to draw the conclusion that the model fits the dataset well [83,84]. Bootstraping method (5000 sample) with bias-corrected percentile method was used to estimate a regression weights, correlations, R-squared value and indirect effect with 95% confidence interval [83,84]. Also, bootstrapping procedures was used to examine the mediation effects [85,86]. Considering that time spent on gaming may be taking into account both as a factor conducive to PVG and as a consequence of this problematic behaviour [87–89], the present study examined two types of models: a model assuming that the number of hours per week spent playing games is the predictor of PVG and a model assuming that amount of time spent on gaming is the result of this problematic behaviour. To examine potential differences in the standardized regression weights and correlations, pairwise parameter comparisons between female (N = 201) and male gamers (N = 169) was used. The critical ratios for differences between parameters with a z-score ≥ 1.96 were considered as significantly different. The same structural equations model for both groups was used [90]. The Statistical calculations were conducted using the statistical software IBM SPSS 23 and AMOS 22.

## Results

The descriptive analysis are presented in the Table 1. In female gamers group, the results of correlation analysis showed a positive correlation between the number of hours per week spent playing games and PVG. The other variables were not correlated with PVG. In male gamers group, a positive correlation between the amount of time spent on gaming, loneliness, PD and PVG was found. Also, there was a negative relationship between self-esteem, self-efficacy and PVG. Detailed findings are presented in Table 1.

**Table 1:** Descriptive statistics and correlations between the variables in female and male gamers group.

Female gamers										
Variables		M	SD	1	2	3	4	5	6	7
1. problematic video gaming		1.52	1.49							
2. game hours		8.00	10.98	0.54***						
3. self-esteem		28.37	5.91	0.12	0.18*					
4. self-efficacy		30.23	4.65	0.02	0.05	0.49***				
5. loneliness		25.43	9.59	0.03	0.01	-0.42***	-0.28***			
empathy	6. empathic concern	41.33	7.42	-0.02	0.01	0.08	0.10	-0.17*		
	7. personal distress	23.95	5.77	0.04	-0.10	-0.43***	-0.49***	0.27***	0.20**	
	8. perspective taking	33.67	5.77	-0.05	0.05	0.04	0.05	0.01	0.44***	0.04
Male gamers										
Variables		M	SD	1	2	3	4	5	6	7
1. problematic video gaming		2.09	1.80							
2. game hours		15.00	15.14	0.39***						
3. self-esteem		29.79	5.93	-0.34***	-0.22**					
4. self-efficacy		30.91	4.72	-0.28***	-0.15	0.57***				
5. loneliness		24.83	9.37	0.29***	0.09	-0.57***	-0.46***			
empathy	6. empathic concern	36.88	7.25	-0.01	-0.12	0.03	0.06	-0.03		
	7. personal distress	21.08	6.07	0.39***	0.05	-0.54***	-0.58***	0.43***	0.15	
	8. perspective taking	33.40	5.72	-0.12	-0.05	0.22**	0.34***	-0.17**	0.42***	-0.25**

***p < 0.001

**p < 0.01

*p < 0.05

**Fig 1:**
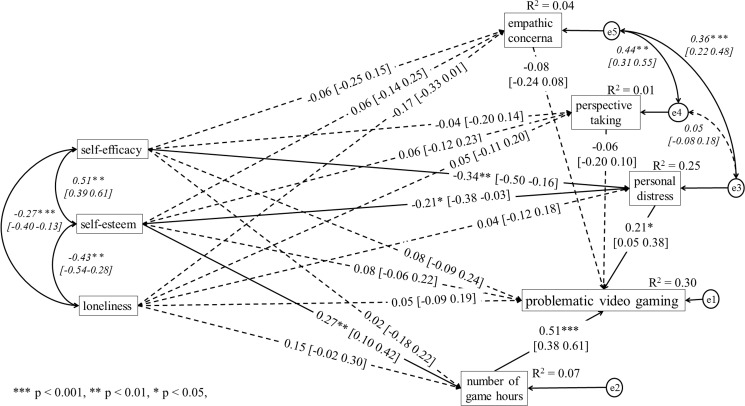
Structural model of relations between the analysed variables in the female group.

**Fig 2:**
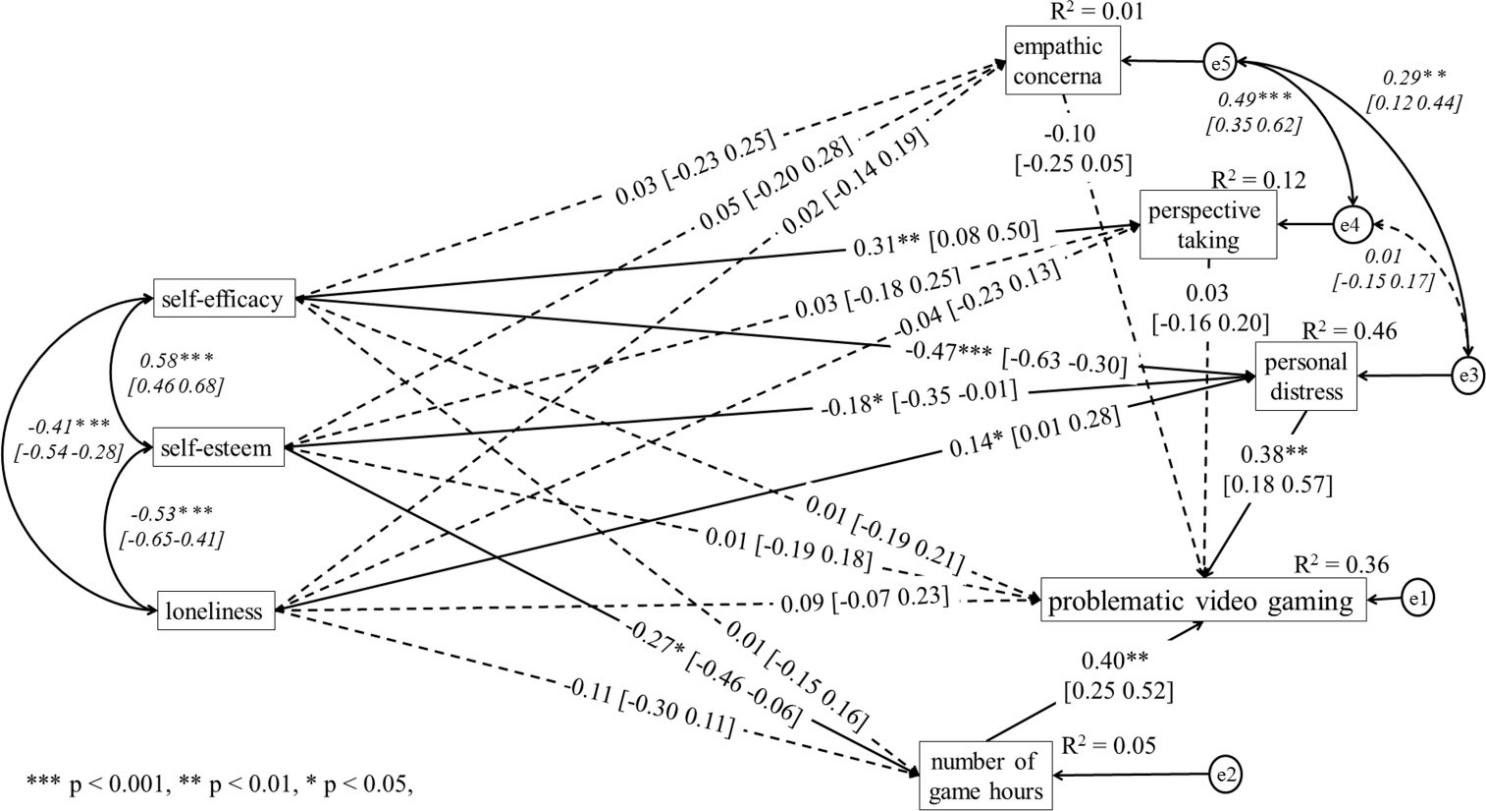
Structural model of relations between the analysed variables in the male group.

Based on the analyses, it was established that the model assuming amount of time spent on gaming as a result of PVG did not well fit the data: χ^2^_(df = 6)_ = 16.91, p = 0.010, χ^2^/df = 2.82; RMSEA = 0.070, SRMR = 0.029, GFI = 0.989, CFI = 0.984, NFI = 0.977, TLI = 0.849. On the other hand, the model assuming the number of hours per week spent playing games as the predictor of PVG was fitted well: χ^2^_(df = 6)_ = 5.90, p = 0.435, χ^2^/df = 0.98; RMSEA = 0.001, SRMR = 0.026, GFI = 0.996, CFI = 1.000, NFI = 0.992, TLI = 1.001. Hence, the model which presupposes that the time of using the game as a predictor of PVG was presented separately for women (see Fig 1) and men group (see Fig 2).

In the female gamers group, there was a positive relation between PD (β = 0.21, p = 0.010, 95%CI: 0.05 0.38), gaming time (β = 0.51, p<0.001, 95%CI: 0.38 0.61) and PVG. No other relation between variables and PVG was found. Furthermore, self-esteem (β = -0.21, p = 0.025, 95%CI: -0.38–0.03) and self-efficacy (β = -0.34, p = 0.001, 95%CI: -0.50–0.16) was negatively related to PD. Also, self-esteem was positively related to gaming time (β = 0.27, p = 0.002, 95%CI: 0.10 0.42). Detailed findings and estimated, standardized regression weights, correlation and their 95% confidence interval (in brackets) are shown in Fig 1.

In the male gamers group, PD (β = 0.38, p = 0.001, 95%CI: 0.18 0.57) and gaming time (β = 0.40, p = 0.001, 95%CI: 0.25 0.52) was positively related to PVG. No other relation between variables and PVG was found. There was a negative relation between self-esteem (β = 0.38, p = 0.001, 95%CI: 0.18 0.57), self-efficacy (β = -0.47, p<0.001, 95%CI: -0.63–0.30) and PD. Also, the positive relation between loneliness and PD (β = 0.14, p = 0.050, 95%CI: 0.01 0.28). The self-efficacy was positively related to perspective taking (β = 0.31, p = 0.007, 95%CI: 0.08 0.50). In opposition to the women group, the negative relation between self-esteem and gaming time was found (β = -0.27, p = 0.014, 95%CI: -0.46–0.06). Detailed findings and estimated, standardized regression weights, correlation and their 95% confidence interval (in brackets) are shown in Fig 2.

The pairwise parameter comparisons showed regression weights differences between female and male gamers in case of relation between the weekly number of hours spent playing games and self-esteem (z = -3.92; p = 0.001). The standardized regression weight was positive in female group (β = 0.27, p = 0.002, 95%CI: 0.10 0.42), whereas it was negative in male group (β = -0.27, p = 0.014, 95%CI: -0.46–0.06). In the case of the relation between self-efficacy and perspective taking, significant differences were found between the analysed groups (z = -2.80; p = 0.005). The path was significant for male group (β = 0.31, p = 0.007, 95%CI: 0.08 0.50), whereas this path was non-significant among female group (β = -0.04, p = 0.652, 95%CI: -0.21 0.14). Moreover, there was difference between female and male gamers in case of relation between loneliness and gaming time (z = -2.04; p = 0.041). However, this relation was non-significant neither in the female (β = 0.15, p = 0.088, 95%CI: -0.02 0.30) nor in the male gamers group (β = -0.11, p = 0.331, 95%CI: -0.30 0.11).

Based on the bootstrapping procedures [59,60], the significant mediation effects of PD was found in female and male gamers group. The self-esteem and self-efficacy exerted a significant indirect effect on PVG via PD. However, the standardized indirect effect of loneliness on PVG mediated by PD was found only in male gamers group. Also, there was a significant indirect effect between PVG and self-esteem via gaming time (see Table 2). This indirect effect had positive sign in female gamers group, whereas it had negative sign in male gamers group. It should be noted that indirect effects with negative sign was probably an example of inconsistent mediation where at least one mediated effect has a different sign than other mediated effect or direct effects [91].

**Table 2:** Bootstrapping standardized indirect effects with 95% confidence intervals in female and male group.

Female gamers					
Model pathways	Point estimates	Standard error	95%CI		p
			Lower	Upper	
self-esteem—personal distress—PVG	-0.072	0.036	-0.164	-0.018	0.007
self-efficacy—personal distress—PVG	-0.044	0.026	-0.116	-0.007	0.015
loneliness—personal distress—PVG	0.007	0.017	-0.020	0.049	0.449
self-esteem–gaming time—PVG	0.137	0.041	0.056	0.219	0.001
Male gamers					
Model pathways	Point estimates	Standard error	95%CI		p
			Lower	Upper	
self-esteem—personal distress—PVG	-0.180	0.062	-0.321	-0.074	0.001
self-efficacy—personal distress—PVG	-0.071	0.039	-0.166	-0.010	0.021
loneliness—personal distress—PVG	0.055	0.027	0.011	0.119	0.022
self-esteem–gaming time—PVG	-0.105	0.046	-0.209	-0.025	0.011

## Discussion

In our study, we focused on investigating such factors as self-esteem, self-efficacy, loneliness, and empathy (understood as Empathic Concern, Personal Distress and Perspective Taking) in a sample of female and male emerging adult video game players. The aim of the study was to look for predictors of PVG among the factors which were considered significant in previous studies about PVG and emerging adulthood: self-esteem, self-efficacy, loneliness and empathy, in a sample of young male and female gamers. We examine whether the relation between self-esteem, self-efficacy, loneliness and PVG is mediated by PD (H1), and whether the relation between PD and PVG exist (H2). Additionally, we verified whether relation between PD and PVG would be stronger in female gamers group than in the male gamers group (H3). And finally, the aim of the study was to check whether, like in the previous studies [7], the time spent playing would be predictor of PVG (H4).

As we hypothesized (H1), the relationship between self-esteem, self-efficacy, loneliness and PVG was fully mediated by PD, and PD was a direct predictor of PVG (H2). With regard to the direct relation between PD and PVG, our findings are in line with previous studies indicating greater PD levels in individuals with behavioral addictions such as gambling addiction [65], problematic Internet use [66] and problematic smartphone use [67] compared to healthy controls. Individuals characterized by higher levels of PD report to be shyer and more socially anxious. Also, PD is associated with deficits in self- and emotion regulation abilities [92,93]. Self-regulation is the ability to manage one’s behaviour instead of being passively affected by external influences. As research demonstrates, in the context of excessive gaming, deficit in self-regulation plays an important role in emergence of problems in personal, professional or academic life, as well as of health problems [94]. PD is a “self-oriented” rather than “other-directed” feeling of personal anxiety, withdrawal, or avoidance instead of an urge to help the suffering person. PD has been negatively related to prosocial behaviour [47,92,95]. Consequently, the results of our research may indicate the importance of interpersonal contacts as a factor conducive to PVG, particularly since Niemz et al. [96] indicate that the Internet can be an alternative form of socialization for individuals who have difficulties maintaining social contacts. Taking into account that emerging adulthood is a developmental stage of many changes in social and working life [17,18], problems in social relations at this stage may lead to the search for alternative forms of social interactions. In this context Maroney et al. [34] found that relationship between psychosocial distress associated with depression, loneliness, social anxiety and PVG was mediated by motivations regarding gaming, such as video game playing to escape negative states and satisfy the needs of social interaction. Therefore, emerging adults players may choose extensive gaming because they have the power to influence their gaming environment and are able to establish interpersonal contacts, which they find difficult outside of the game environment and which constitutes a source of negative experiences for them [3].

However, it should be noted that the relationship between loneliness and PVG was no mediated by PD in female gamers group. One of the possible explanations is related to the fact that females tend to have more cohesive relationships and closer to their social relationships than males [97]. In this context, Von Der Heiden [22] showed that male players are more likely to choose video games for social networking reasons than female players. Additionally, Morahan-Martin and Schumacher [98] showed that lonely people use the internet for emotional support more than people who are less lonely. In this context, female, compared to male users, spend more time on social network sites than they expected, feel more close to friends on social networking sites than to everyday life, and more dependent on this medium [99]. Consequently, loneliness may be less related to the well-being of female than male gamers, because female gamers may experience greater online as well as off-line support. It also should be noted that females used social support-seeking coping strategy more often than males [35]. Additionally, emerging adult females reported better friendship quality then emerging adult males [100]. Consequently, adult women can cope better with social change during their youth than adult men. In our SEM models, PD full mediation effect between self-esteem, self-efficacy, loneliness and PVG was observed. One of the explanations is that PD may be a construct which combines all of the above-mentioned factors such as self-esteem, loneliness and self-efficacy. Previous studies also indicate a relationship between self-esteem, loneliness, self-efficacy and PD [47–50]. Given that PD is a complex self-oriented emotional reaction of personal anxiety [47], and that levels of self-esteem, loneliness and self-efficacy are formed early in life [101], it can be supposed that low self-esteem, low self-efficacy and high loneliness may be the factors leading to the development of PD in emerging adulthood. Our findings suggest that PD, as a function of self-esteem, loneliness and self-efficacy, is an important predictor of PVG. This relationship was not found in previous studies due the specificity of the analyses of PVG predictors [70]. In most cases, only individual predictors were analysed, and relationships between the predictors were not considered in regression models.

In both groups, we confirmed H4 that assumed the number of hours spent playing video games per week is a predictor of PVG. Our results support evidence from previous research in terms of the impact of the time devoted to video games on PVG [102]. Przybylski and colleagues [103] showed that an increase in the weekly number of hours dedicated to video games leads to a decrease in the level of life satisfaction for people with symptoms of PVG. However, the connection between PVG and the time spent on video games must be approached with caution because some other findings indicate that the latter is a poor predictor of problematic behaviour [104].

Contradictory to our assumptions (H3), we no found difference between female and male emerging adult gamers in case of the relation between PD and PVG. Our results may indicate that, despite the differences in the level of PD [63,64], the self-oriented feelings of personal anxiety and unease in intense interpersonal settings [47] was important predictor of PVG in female and male gamers. However, it should be noted that PD was associated with low self-esteem, low self-efficacy and high loneliness in male gamers group, whereas it was associated with low self-esteem and low self-efficacy in female gamers group. Consequently, PD may different characteristic in both groups, which no change the fact that PD may be relevant predictor of PVG.

Additionally, we presented differences between female and male gamers in case of the relation between the weekly number of hours spent playing games and self-esteem. The standardized regression weight was positive in female gamers group, whereas it was negative in male gamers group. One of the possible explanations may be related to a different function of playing games in female and male gamers. Chou and Tsai [58] showed that male compare to female gamers had stronger motivation to game playing, which was associated with entertainment, seeking information and social device. Also, in male gamers, the increased playing time may lead to increase feelings of success and achievement, which then provide to increase playing time [105]. According to model of compensatory Internet use [106], PVG may be more related to compensation. In particular, individuals who cannot overcome certain life situations and find it difficult to meet their needs in the real world may be motivated to use the Internet to relieve negative emotions and compensate for needs in the virtual world. In this context, the low self-esteem, associated with negative feelings about oneself, may lead to increased playing time, which may provide to increase feelings of success and achievement in male gamers [105]. Consequently, low self-esteem may contribute to more hours of playing games in male gamers. However, in female compare to male gamers group, we showed opposite relation between self-esteem and playing time. Taking into account that games are mostly created for players [31], female gamers probably they must have greater self-esteem to enter game world most players are male. It should be noted that females may probably compensate for needs associated with low self-esteem in the virtual world, however they can choose other applications, e.g. social networking site for it [99].

### Limitations and future study

There are some limitations to our research which should be taken into consideration when discussing the results. Our participants were only young individuals, who constitute the majority of players. However, further research needs to establish whether similar relationships between PVG and psychological factors can be identified in other groups of players like children, adolescents or seniors etc. Also, the research was conducted on a Polish sample. Therefore, further multicultural research is needed to determine whether similar relationships can be found in participants from other countries. Recent research has highlighted the important role played by genetic and epigenetic factors in the onset and maintenance of PVG [107]. However, the possible role genetic risk factors in the onset and maintenance of PVG was not included in this current research. Consequently, future PVG research into the complete biopsychosocial model is needed. In the future, research on PVG should also focus more on analysing the models which take into account the relationships between the predictors of PVG than on analyses of individual predictors of this phenomenon.

## Supporting information

S1 Dataset Data base of study. (CSV)
